# Case Report: A Rare Case of Nasal Forehead Mass in Kimura's Disease

**DOI:** 10.3389/fsurg.2021.672291

**Published:** 2021-05-21

**Authors:** He Zhao, Zhi-wei Cao, Zhao-wei Gu

**Affiliations:** Department of Otolaryngology Head and Neck Surgery, Shengjing Hospital of China Medical University, Shenyang, China

**Keywords:** Kimura disease, surgery, nasal forehead mass, case report, prednisone

## Abstract

**Background:** Kimura's disease is a rheumatic immune disease and head and neck lymph nodes are often involved. A mass occurring in the nasal forehead is rare. Good prognosis after surgical resection by glucocorticoid therapy is more rare.

**Case Summary:** We report the rare case of a nasal forehead mass in a 45-year-old male patient with Kimura's disease. The patient underwent resection of the mass in October 2018 in a local hospital and the postoperative pathology was unclear. He then underwent a second resection in our department in December 2019 mainly because growth of the mass was affecting his appearance. Postoperative pathology confirmed that the patient had Kimura's disease, and he accepted systemic treatment with prednisone. We followed the patient for 10 months after surgery. He is now recovering well and continues to be closely monitored during follow-up.

**Conclusion:** It is rare that the painless mass in the nasal forehead is diagnosed as a Kimura's disease.After completely resection of the mass and systemic treatment with prednisone, the patient had a good outcome. We provide experience for the treatment of Kimura's disease in nasal forehead.

## Introduction

Kimura's disease is a rare, chronic inflammatory disease of unknown etiology that occurs rarely in the nose and often affects the lymph nodes. Its clinical manifestations vary according to the location and size of the masses, which are mainly painless with progressive growth (1). Recently, a patient with Kimura's disease of the forehead above the nose was admitted to our ward. The patient's clinical data are described.

## Case Presentation

### Chief Complaints

A 45-year-old male patient presented with complaints of a nasal forehead mass which had gradually increased in size over the past 5 years and influenced his appearance.

### History of Present Illness

The patient found the nasal-root mass 5 years ago. The mass was the size of a peanut, soft, of normal skin color and with a swollen smooth surface, often exhibiting self-absorption with no other discomfort. In October 2018, nasal mass resection was performed at a local hospital and postoperative pathology was unclear. A relapse occurred in January 2019 and the mass gradually increased in size over the next 10 months, to approximately the size of 5 cm × 2.5 cm. The patient came to our department for a clear diagnosis and treatment.

### History of Past Illness

The patient was in good health and had no history of chronic disease. In 2012, he underwent appendectomy in a local hospital due to acute appendicitis.

### Physical Examination

Physical examination of the nose upon admission showed that the shape of the nose was normal. The left part of the nasal-root mass was about the size of 5 cm × 2.5 cm. It was soft, with a smooth surface, no redness or swelling, no ulceration, and no tenderness.

### Laboratory Examinations

No abnormalities in terms of renal function, erythrocyte sedimentation rate (ESR), rheumatism, serum complement (C3/C4), quantitative immunoglobulin determination, immunoglobulin IgG4, antinuclear antibodies or ANCA were found. The possibility of Kimura's disease was not considered before surgery, and IgG4 and IgE levels were not detected before the operation. Positive laboratory results are shown in Table 1.

**Table 1:** Positive laboratory results.

**Test**	**Before surgery**	**1 month after surgery**	**4 months after surgery**	**10 months after surgery**
Percentage of eosinophils (Routine blood test)	27.6%	21.4%	3.2%	1.6%
Eosinophils (Routine blood test)	2.0 × 10^9^/L	1.7 × 10^9^/L	0.3 × 10^9^/L	0.2 × 10^9^/L
IgG4n	Not detected	0.857 g/L	0.553 g/L	0.368 g/L
IgE	Not detected	>2,500 IU/mL	>824 U/mL	>764 IU/mL

### Imaging Examinations

**Figure 1:**
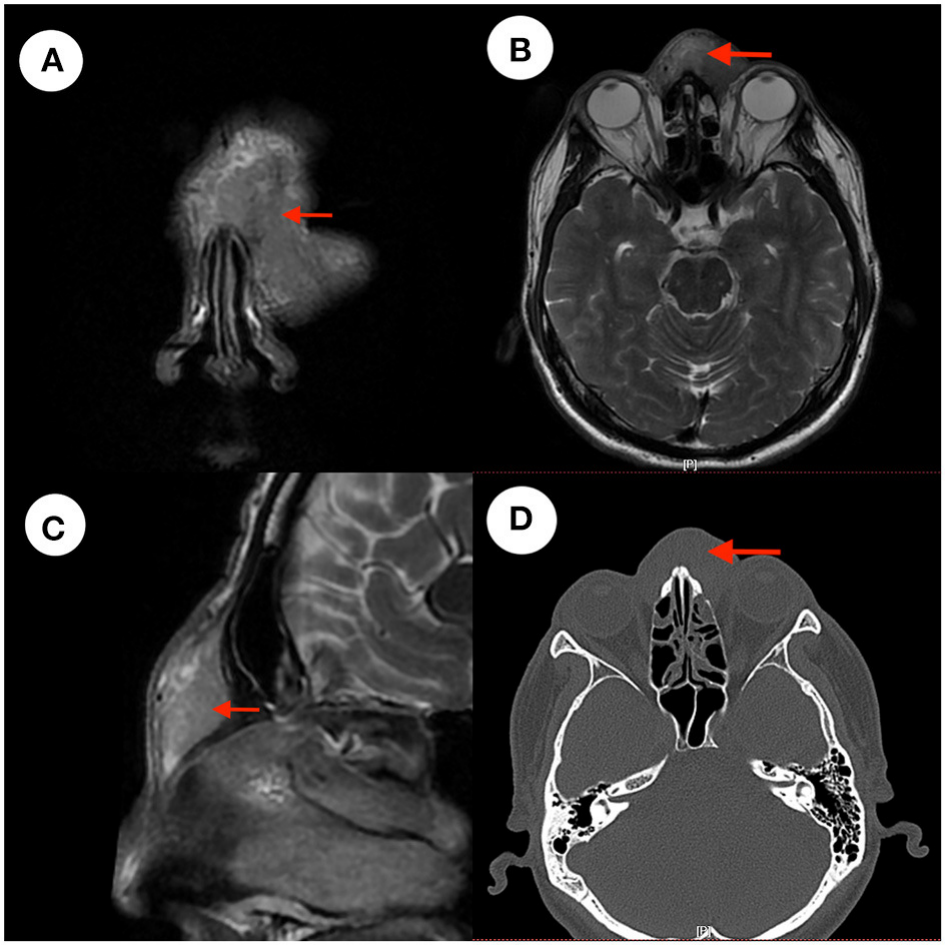
CT and MRI images. **(A–C)** Show iso- and hyper-intense signal in T2 sequence of the patient's cranium MRI images. Panel **(A)** is a coronal T2 image. Panel **(B)** is an axial T2 image. Panel **(C)** is a sagittal T2 image. Panel **(D)** is a CT image, with the red arrow indicating the mass.

After admission, computerized tomography (CT) and magnetic resonance imaging (MRI) examination of the patient's paranasal sinuses were performed. MRI showed an iso-intense signal in the T1 sequence, iso- and hyper-intense signal in the T2 sequence (Figures 1A–C) and enhancement in the T1 enhanced sequence with an unclear boundary. CT showed a soft tissue signal in the nasal forehead with an unclear boundary (Figure 1D).

### Pathological Examinations

**Figure 2:**
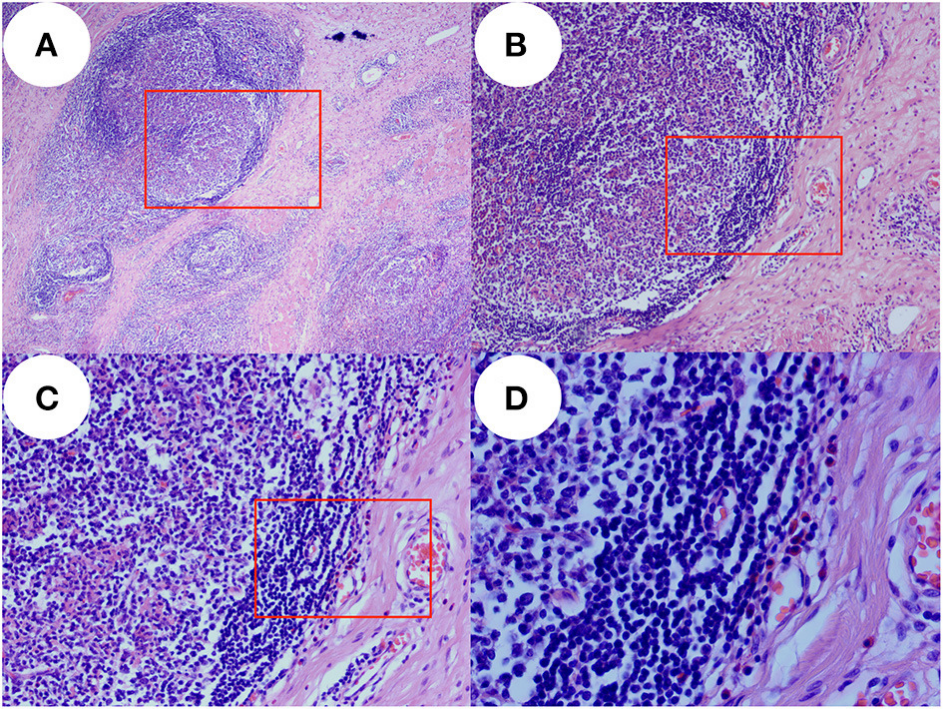
Pathology images. In the proliferative fibrous tissue, there were proliferative lymphoid tissue, lymphoid follicles, a large number of eosinophils between the follicles, proliferative small vessels in the follicles, and local infiltration of striated muscle. Panel **(A)** is pathological images taken with an optical microscope at 40× magnification and image **(B)** was taken at red rectangle. Panel **(B)** is pathological images taken with an optical microscope at 100× magnification and image **(C)** was taken at red rectangle. Panel **(C)** is pathological images taken with an optical microscope at 200× magnification and image **(D)** was taken at red rectangle. Panel **(D)** is pathological images taken with an optical microscope at 400× magnification which show many eosinophils in the tissue.

The pathological findings were as follows: in the hyperplastic fibrous tissue, there were hyperplastic lymphoid tissues, lymphoid follicles, a large number of eosinophils between follicles and small hyperplastic blood vessels in the follicles, which focally infiltrated striated muscle (Figure 2). Immunohistochemistry findings were as follows: CD3(+), CD20(+), CD21(FDC+), CK(–), and Cyclin D1(–).

## Final Diagnosis

Kimura's disease of the nasal forehead.

## Treatment

**Figure 3:**
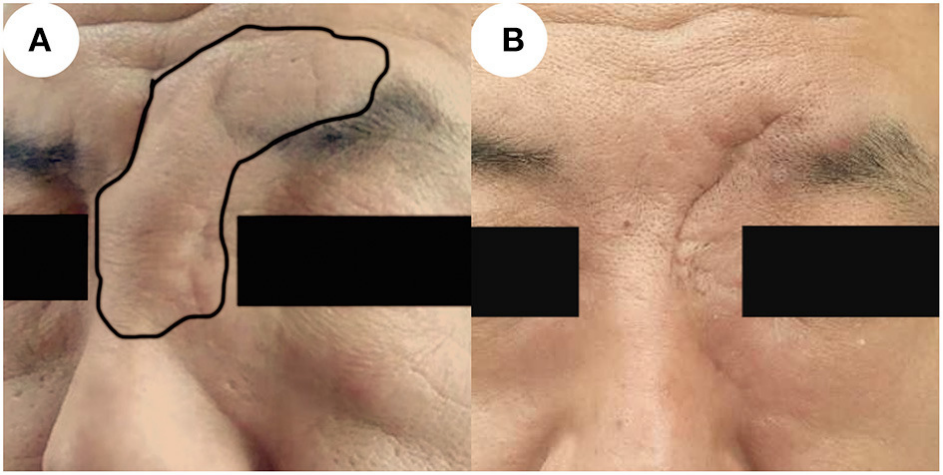
Images before and after surgery. **(A)** Photo taken prior to surgery, showing the local updrift at the nasal forehead which in the black circle. **(B)** Six months after surgery, the area is of normal appearance without recurrence.

The patient underwent surgical decompression of the nasal forehead mass under general anesthesia in December 2019, and then accepted treatment with prednisone following the diagnosis of Kimura's disease. After skin incision, subcutaneous solid tumor could be seen, and the capsule was clear and complete. The tumor was completely removed along the tumor capsule, and the operation cavity was checked, and no residue was found. The surface projection is shown in Figure 3. One month after operation, he was given prednisone 30 mg once a day for 4 weeks and 20 mg once a day for 4 weeks and 10 mg once a day for 4 weeks, total 12 weeks to control the disease, and supplemented with pantoprazole to protect the stomach and a calcium supplement.

## Outcome and Follow-up

Subcutaneous effusion occurred in the postoperative period and recovered after 1 week. There was no recurrence 6 and 10 months after surgery, and the appearance of the nose and forehead recovered well with acceptable scarring. The relevant images are shown in Figure 3.

## Discussion

Kimura's disease is a rare and benign immune-system disease of unknown etiology that occurs particularly in the maxillofacial region. It is characterized by lympho-proliferative formation of lymphoid follicles and eosinophilic granulomas in soft tissue or lymph nodes (2). Kimura's disease may occur at any age, although the peak age of occurrence is 20–40 years; the majority of cases occur in middle-aged males (3, 4). The patient reported in this article was a 45-year-old male. The first onset of the disease was 5 years ago, after which he underwent resection of the mass in 2018. Kimura's disease should be differentiated from the following diseases: benign tumors, lymphomas, Kaposi sarcoma, and Langerhans cell histiocytosis.

At present, the diagnosis of Kimura's disease depends mainly upon histopathology. CT and MRI are also helpful in the diagnosis of Kimura's disease. Most lesions demonstrate mild or moderate enhancement in post-contrast CT scans and moderate or marked enhancement in post-contrast MR images (5). Tissue infiltration, lymphocyte follicular hyperplasia, fibro-collagenous deposition, and vascular proliferation are the histopathological characteristics of Kimura's disease (6).

In the past, Kimura's disease was sometimes confused with ALHE (angiolymphoid hyperplasia with eosinophilia). ALHE occurs predominantly in the skin, as opposed to Kimura's disease, which occurs as a subcutaneous lesion. Both are characterized by heavy eosinophilic infiltration, vascular proliferation and a predilection for appearing in the head and neck region. Conspicuous features of Kimura's disease on histopathology include hyperplastic lymphoid follicles with prominent germinal centers and eosinophilic infiltration. Laboratory tests show that eosinophils on routine blood testing and IgE in serum are significantly increased in patients with Kimura's disease (6–11). Sometimes, Kimura's disease can occur in other organs, especially the kidneys, where it takes the form of membranoproliferative glomerulonephritis (12, 13). The eosinophils on routine blood testing and IgE in serum are significantly increased, and the diagnosis of Kimura's disease is certified without ALHE.

At present, there is no standard guideline or consensus for the treatment of Kimura's disease. The main treatment for Kimura's disease is surgical removal of the mass and pathological examination, and continue treatment after surgery. According to previous literature, there are hormone therapy, radiotherapy, immunotherapy and treatment of ischemic vascular reconstruction (14, 15). For those masses that cannot be completely removed, postoperative radiotherapy is more important. Research has shown that surgical excision combined with low-dose radiotherapy shows better results than surgical excision or radiotherapy alone (16). Because the boundary of the mass was clear and the mass was completely removed during the operation, we did not give the patient radiotherapy after the operation.

Local or systemic steroids can significantly reduce the mass and control the lesions, lymphadenopathy and nephrotic syndrome (17). Oral prednisolone has a high degree of individualization, and administered in different doses, durations and gradual reduction schemes according to the size of the primary or recurrent lesions (18–20). Even so, the mass also has recurrence after glucocorticoid reduction (about 45.8%) (21).

Due to the higher eosinophils and IgE in the peripheral blood of the patients, although the mass has been completely removed, after comprehensive consideration, we give the patients oral glucocorticoid therapy, showing a stepwise decline.

Although recurrence is common, no significant recurrence was observed during follow-up in this case. After systemic treatment, eosinophils on routine blood testing and IgE in serum decreased gradually especially the eosinophils which reached normal values. We believe that systemic drug therapy controls eosinophil and IgE levels, and is an important method for preventing postoperative recurrence which the mass was removed completely with high eosinophils and IgE in the peripheral blood.

## Conclusion

Kimura's disease is rare and is mainly diagnosed based on histological examination, as well as physical and radiological examination. Outcome is significantly better after systemic therapy. In the present case, although no recurrence was noted during the 10-month follow-up period, further monitoring is required. Our study demonstrates that a painless nasal forehead mass should be excluded from Kimura's disease and nasal forehead Kimura's disease has a good outcome after surgery and prednisone treatment without radiotherapy.

## Data Availability Statement

The raw data supporting the conclusions of this article will be made available by the authors, without undue reservation.

## Ethics Statement

The studies involving human participants were reviewed and approved by Ethics Committee of Shengjing Hospital. The patients/participants provided their written informed consent to participate in this study. Written informed consent was obtained from the individual(s) for the publication of any potentially identifiable images or data included in this article. Informed written consent was obtained from the patient for publication of this report and any accompanying images.

## Author's Note

The authors have read the CARE Checklist (2016), and the manuscript was prepared and revised according to the CARE Checklist (2016).

## Author Contributions

HZ, Z-wG, and Z-wC reviewed the literature and contributed to manuscript drafting. Z-wG was responsible for revision of the manuscript for important intellectual content. All authors issued final approval for the version to be submitted.

## Conflict of Interest

The authors declare that the research was conducted in the absence of any commercial or financial relationships that could be construed as a potential conflict of interest.
